# Pork Quality and Expression of Genes Involved in Muscularity and Fat Deposition in Different Commercial Lines and Sexes of Pigs

**DOI:** 10.3390/ani15233363

**Published:** 2025-11-21

**Authors:** Julia Dezen Gomes, Bruna Pereira Martins da Silva, Stefano Francisco Pereira Duarte, Soraia Viana Ferreira, Fernanda Nery Ciconello, Vivian Vezzoni de Almeida, Laura Woigt Pian, Cristina Tschorny Moncau-Gadbem, Mônica Corrêa Ledur, Matheus Emanuel Malaquias, Júlio César de Carvalho Balieiro, Aline Silva Mello Cesar

**Affiliations:** 1 Luiz de Queiroz College of Agriculture, University of São Paulo, Avenue Pádua Dias 11, Piracicaba 13418-900, São Paulo, Brazil; juliadezen@usp.br (J.D.G.); brunamartins@usp.br (B.P.M.d.S.); stefanoduarte@usp.br (S.F.P.D.); fernanda.ciconello@usp.br (F.N.C.); laura.woigt@usp.br (L.W.P.); matheusmalaquias@usp.br (M.E.M.); 2 Danbred Brasil, Avenue Juscelino Kubitschek de Oliveira 2094, Patos de Minas 38706-000, Minas Gerais, Brazil; soraia@db.agr.br; 3 School of Veterinary Medicine and Animal Science, Federal University of Goiás, Chácaras Califórnia, Goiânia 74691-835, Goiás, Brazil; vivian.almeida@ufg.br; 4 Department of Nutrition and Animal Production, School of Veterinary Medicine and Animal Science, University of São Paulo, Avenue Duque de Caxias Norte 225, Pirassununga 13635-900, São Paulo, Brazil; crismoncau@gmail.com (C.T.M.-G.); balieiro@usp.br (J.C.d.C.B.); 5 Embrapa Suínos e Aves, Highway BR-153, Km 110, Tamanduá District, Concórdia 89715-899, Santa Catarina, Brazil; monica.ledur@embrapa.br

**Keywords:** gene expression, meat quality, pig breeding, Duroc, Pietrain

## Abstract

Pork is a major source of animal protein worldwide, and its quality is influenced by pre- and post-slaughter procedures. Advances in molecular biology, particularly gene expression studies, support genetic improvement programs by enabling precise strategies to enhance meat quality and economic sustainability. This study evaluated meat quality traits and candidate gene expression in muscle and subcutaneous adipose tissue from different genetic lineages and sexes. A total of 120 pigs from three lineages—Line D (½ Duroc × ½ DB90), Line P (½ Pietrain × ½ DB90), and Line H (½ [Duroc and Pietrain] × ½ DB90); including immunocastrated males (IM) and females, were randomly selected. Meat quality was assessed using physicochemical parameters, and gene expression analysis was performed in 36 pigs using RT-qPCR with *B2M*, *TBP*, and *RPL4* as references, and *COL1A1*, *PRKAR2A*, *CAST*, *ADIPOQ*, and PPARGC1A as targets. Lineage influenced drip loss and intramuscular fat, while lineage–sex interaction affected tenderness and color (L* and b*), and sex influenced b*. In muscle, sex affected *COL1A1* and *PRKAR2A*, and lineage influenced *COL1A1* and *CAST*. In adipose tissue, only *CAST* was lineage-dependent.

## Simple Summary

This study evaluated pork quality traits and candidate gene expression across different genetic lineages and sexes. A total of 120 pigs from three lineages, including immunocastrated males and females, were analyzed. Meat quality was assessed through physicochemical parameters, and gene expression was measured in 36 pigs using RT-qPCR. Lineage affected drip loss and intramuscular fat, while lineage–sex interactions influenced tenderness and color (L* and b*), and sex affected b*. In muscle, sex influenced *COL1A1* and *PRKAR2A* expression, and lineage affected *COL1A1* and *CAST*. In adipose tissue, only *CAST* expression was lineage-dependent. These findings provide insights for genetic strategies to improve pork quality.

## 1. Introduction

Pork is one of the main sources of animal protein consumed globally and represents a key component of the world’s agri-food economy [1,2]. The growing consumer demand for high-quality meat has driven scientific efforts to enhance traits that directly influence consumer acceptance and purchasing decisions.

Pork meat quality is a multifactorial trait shaped by a complex interplay between pre-slaughter conditions and postmortem processes. These interactions can act synergistically or antagonistically, affecting key sensory attributes such as color, tenderness, juiciness, and flavor, the main determinants of consumer perception and market value [3]. Beyond these sensory factors, sex effect is a critical biological variable that significantly affects meat quality [4,5]. Its influence extends beyond compounds associated with boar taint, encompassing broader effects on meat and fat composition that ultimately shape consumer acceptance [4].

Sex-related differences in pig body composition are associated with variations in protein and fat deposition, modulated by hormonal regulation [6]. Androgenic hormones, such as testosterone, and estrogens regulate energy metabolism and tissue distribution. In immunocastrated males, the administration of the second vaccine dose leads to a decline in testosterone levels, resulting in increased fat deposition and alterations in meat composition [7,8,9]. In contrast, females are primarily influenced by estrogens, which result in lower fat accumulation and greater lean meat deposition [7,8,9]. These hormonal influences influence park characteristics such as tenderness and water-holding capacity, thereby impacting overall product quality.

In addition to the sex effect, genotype exerts a substantial influence on meat quality, as breed selection remains a key strategy to enhance organoleptic and nutritional properties. The Duroc breed, for instance, is often recognized for contributing positively to meat sensory quality, particularly due to its high intramuscular fat content, which directly influences tenderness and flavor [3]. Such breed-related differences highlight the intricate interaction between genetic background and production environment [3].

In this context, the selection of terminal sire lines has emerged as an effective approach to improve meat quality while maintaining economic efficiency [10]. This strategy enables producers to meet evolving market demands that increasingly emphasize superior flavor, texture, and nutritional composition.

Among the strategies employed in genetic improvement, the identification and study of genes associated with meat quality, such as *CAST* (Calpastatin), *COL1A1* (Collagen Type I Alpha 1 Chain), and *PRKAR2A* (Protein Kinase cAMP-Dependent Type II Regulatory Subunit Alpha), have provided valuable molecular markers to guide selection programs [11,12,13]. These genes participate in physiological pathways affecting texture, water retention, and fat deposition, all of which are critical for consumer satisfaction. As a result, the selection of favorable gene variants can significantly impact tenderness, juiciness, and fat content, ultimately affecting the flavor and appearance of the meat.

The use of these expression biomarkers in breeding programs offers a powerful means to assess how genetic and environmental factors shape complex phenotypes like meat quality [14,15]. Studies have examined gene expression associated with key pork traits, deepening understanding of the biological mechanisms underlying these characteristics and enabling progress in both genetic selection and product quality improvement [14,15,16].

Thus, the advancement of knowledge in molecular biology, especially gene expression, plays a crucial role in the genetic improvement of pigs. A detailed understanding of the molecular mechanisms involved in the expression of genes related to traits of interest, such as meat quality and production efficiency, enables the development of more precise and effective strategies in breeding programs [17]. The integration of these tools with traditional management and nutrition practices can enhance the efficiency of improvement programs, reduce costs, and promote greater economic and environmental sustainability within the global swine industry [16,18].

Based on this context, we hypothesize that pork quality is modulated by the interaction between genetic lineage and sex, influencing both physicochemical properties and the expression of candidate genes in muscle and subcutaneous adipose tissue. Therefore, this study aimed to evaluate pork quality traits and investigate the expression of selected candidate genes across different genetic lines and sexes to better understand how these factors collectively determine meat quality.

## 2. Materials and Methods

Animal procedures were conducted and performed in accordance with the Ethics Committee on Animal Use of the “Luiz de Queiroz” College of Agriculture (University of São Paulo, Piracicaba, Brazil, CEUA protocol number: 7416051222).

### 2.1. Animals and Experimental Design

The study was conducted at a commercial, located in the municipality of Varjão de Minas, in the State of Minas Gerais, Southeast region of Brazil. The area is situated at an altitude of 842 m, with coordinates of 18°34′ South latitude and 46°31′ West longitude, and features a tropical savanna climate with a dry winter season, according to the Köppen climate classification (Aw).

The experimental swine facility was equipped with natural lighting and a ventilation system, and pens with partially slatted concrete floors. Animals had free access to feed and water throughout the 106-day feeding study.

The animals belonged to three commercial genetic lines, differentiated by the sire used in each crossbreeding. All animals shared the same maternal origin, DanBred Hybrid, resulting from the cross between the Yorkshire and Landrace breeds. The male breeder lines corresponded to the experimental treatments: Line D—Duroc, Line H—Hybrid (Duroc and Pietrain), and Line P—Pietrain. In total, 600 pigs were selected for the experiment, comprising 100 males and 100 females from each cross, as previously described by our group in Gomes et al. (2025) [19].

The entire males were subjected to immunocastration, which was performed by administering two 2 mL doses of Vivax^®^ (Pfizer Animal Health, Parkville, Australia). These doses were given at 105 and 133 days of age, following the manufacturer’s guidelines.

During each phase of the study, all pigs were fed the same diet, which was corn- and soybean meal-based and formulated to meet or exceed all nutrient requirements for growing and finishing pigs [20]. The diets for the growing pig phase were formulated to 1.10% standardized ileal digestible (SID) lysine (Lys) and 3.300 kcal/kg of metabolizable energy (ME), whereas the diet formulation included 0.98% SID Lys and 3.320 kcal/kg of ME for the finishing pig phase.

### 2.2. Slaughter and Sampling Procedures

At the end of the study, at 169 days of age, a subset of 120 pigs was selected from the original group of 600 animals as described in our previous study [19]. Selection was based on body weight, and animals with body weights within ±5% of the pen average closest to the pen average were selected (*n* = 120), comprising 20 pigs per treatment (three genetic lines and two sexes). The pigs were transported to a commercial slaughterhouse (Suinco, Patos de Minas, MG, Brazil), where, following a 16-h fast from solid feed with free access to water, they were subjected to electrical stunning followed by exsanguination according to normal industry procedures. After slaughter, carcasses were chilled at 4 °C for 24 h, at which time a 2.5-cm chop of *Longissimus lumborum* (LL) muscle was removed from the left sides between the 9th and 10th ribs.

### 2.3. Pork Quality Analysis

**Figure 1:**
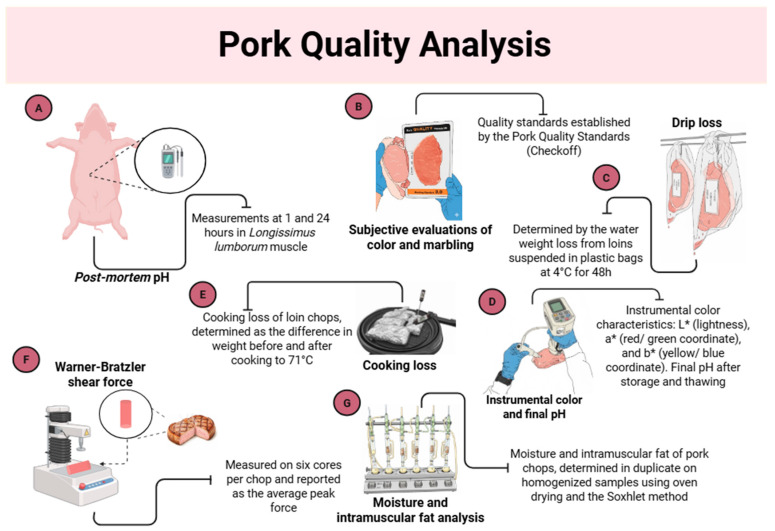
Illustrative scheme of pork quality analyses performed in the *Longissimus lumborum* muscle: (**A**) post-mortem pH; (**B**) subjective evaluation of color and marbling; (**C**) drip loss; (**D**) instrumental color and final pH; (**E**) cooking loss; (**F**) Warner-Bratzler shear force; and (**G**) moisture and intramuscular fat analyses.

Meat quality analyses are illustrated in Figure 1. Post-mortem pH measurements at 1 and 24 h in LL muscle were performed using a HI-98163 pH meter with a stainless-steel probe (Hanna Instruments, Woonsocket, RI, USA).

Subsequently, two loin chops had external fat and connective tissue removed according to the commercial standards of the slaughterhouse, and were deboned. At the time of deboning, a trained professional performed subjective evaluations of color and marbling on each pork sample, using the quality standards established by the Pork Quality Standards (Checkoff), National Pork Board, Des Moines, IA, USA (2010). The surface color of the LL muscle was visually compared with a standard scale ranging from 1 (lightest) to 6 (darkest). Similarly, marbling, referring to the amount of visible intramuscular fat, was assessed using a standard scale ranging from 1 (least marbling) to 10 (most marbling).

The first loin chop was used for drip loss analysis, where a chop from each pig was initially weighed and suspended on a hook inside an inflated and sealed plastic bag. The loins were stored in a refrigerator at 4 °C for 48 h. At the end of this period, they were reweighed, and drip loss was calculated as the difference between the final and initial weights [21]. A second loin chop from each animal was obtained for the remaining analyses of meat quality.

Instrumental color characteristics, L* (lightness), a* (red/green coordinate), and b* (yellow/blue coordinate), were measured at three different points on the steaks from each animal using a Konica Minolta CR-400 digital colorimeter (Konica Minolta Sensing Americas, Inc., Ramsey, NJ, USA), calibrated according to the manufacturer’s settings (D65, Y = 93.7, X = 0.3160, y = 0.3323). Final pH after storage and thawing was measured again using a HI-98163 pH meter with a stainless-steel probe (Hanna Instruments, Woonsocket, RI, USA).

For the analysis of cooking loss, a previously thawed loin chop was used, which was weighed following the procedures described by AMSA (2015) [22]. The loin chops were wrapped in aluminum foil and placed on a heating plate until reaching an internal temperature of 71 °C at the geometric center, monitored using a portable digital thermometer (TP101, HM-600, Tatuapé, São Paulo, SP, Brazil) [22]. After removal from the heating plate, the loins were allowed to cool at room temperature and then reweighed. Cooking loss was calculated as the difference between the weights before and after cooking.

The Warner-Bratzler shear force was measured as described by Honikel (1998) [21]. For this analysis, the same loin chops used in the cooking loss test, previously cooled overnight at 4 °C, were utilized. Six cores, each with a diameter of 1.27 cm, were cut parallel to the muscle fiber direction from each chop. These cores were sheared once through the center using a Warner-Bratzler ‘V slot blade’ (Warner-Bratzler meat blade, Macmesin BFG 500N; GR Manufacturing Co., Collins LN, Manhattan, KS, USA), and the Warner-Bratzler shear force was recorded as the peak force on the curve (N/s). The shear force value for each sample was reported as the average of the six cores.

Finally, a third pork chop was homogenized using a commercial processor for moisture and intramuscular fat analyses, performed in duplicate. For these analyses, 5 g of each homogenized steak was dried in a ventilated oven at 105 °C for 12 h. After drying, the sample was weighed to calculate the moisture content. The same sample was then used for intramuscular fat analysis by the Soxhlet method [23]. In this method, a Soxhlet apparatus was employed, where hexane was heated in a flat-bottom flask, vaporized, and condensed in a condenser. The accumulated extract was siphoned back into the flask, and the process was repeated cyclically for 6 h to obtain the intramuscular fat.

### 2.4. Tissue Collection and RNA Extraction

After exsanguination, among the 120 animals selected for meat quality, 36 left half-carcasses (*n* = 36) were randomly chosen for sampling of the LL muscle and subcutaneous adipose tissue adjacent to the loin for subsequent total RNA extraction. A total of 12 samples per line were selected, including IM and females (F). Approximately 200 mg of each tissue was carefully collected under conditions that prevent RNA degradation and preserved in RNALater^®^ (Thermo Fisher Scientific Inc., Waltham, MA, USA), then stored at −20 °C until analysis.

Total RNA extraction was performed using approximately 100 mg of each tissue. The tissue was pulverized in liquid nitrogen, and total RNA was extracted using 1 mL of Trizol^®^ reagent (Invitrogen, Inc., Carlsbad, CA, USA) and 200 µL of chloroform, resulting in phase separation into three layers: the upper aqueous phase (clear), containing the RNA; the interphase (white), containing chloroform, DNA, and proteins; and the lower organic phase (reddish), corresponding to phenol. Subsequently, the commercial PureLink™ RNA Mini Kit (Invitrogen, Inc., Carlsbad, CA, USA) was used following the manufacturer’s instructions, and samples were stored at −80 °C until gene expression analyses.

### 2.5. Quantification and Integrity of Total RNA

The RNA concentration for each sample was determined using a NanoDrop spectrophotometer (Thermo Fisher Scientific Inc.) with 1 µL of total RNA. Sample purity was assessed by the ratio of absorbance measured at wavelengths of 260 nm (A260) and 280 nm (A280). All extracted RNA samples showed an A260/A280 ratio between 1.8 and 2.0, indicating high purity. The integrity of total RNA was verified using the Bioanalyzer 2100 (Agilent, Santa Clara, CA, USA). All RNA samples presented an RNA Integrity Number (RIN) greater than eight.

### 2.6. Gene Expression Analysis

Five candidate genes were selected based on previously reported evidence in the scientific literature highlighting their involvement in meet quality traits to be evaluated for their gene expression profile in the muscle and adipose tissues: PPARG coactivator 1 alpha (*PPARGC1A*), Collagen type I alpha 1 chain (*COL1A1*), Protein kinase cAMP-dependent type II regulatory subunit alpha (*PRKAR2A*), Calpastatin (*CAST*), and Adiponectin, C1Q and collagen domain containing (*ADIPOQ*) [11,13,24,25,26].

To ensure a precise and reproducible quantification of these target transcripts, a targeted RT-qPCR (quantitative reverse transcription PCR) approach was employed, enabling accurate assessment of relative gene expression levels associated with meat quality phenotypes. RNA concentrations were standardized to 20 ng/µL to ensure consistency in the subsequent step. Samples with higher concentrations were diluted using nuclease-free water. Gene expression analysis was performed using RT-qPCR with the commercial qPCRBIO SyGreen 1-Step Go Lo-ROX kit (PCR Biosystems Inc., Archway Road, London, UK). Primer sequences are listed in Supplementary Table S1. Each reaction was prepared using 5 µL of 2× PCRBIO SyGreen 1-Step Mix (PCR Biosystems Inc., Archway Road, London, UK), 0.4 µL of forward primer (10 µM), 0.4 µL of reverse primer (10 µM), 0.5 µL of 20× RTase Go (PCR Biosystems Inc., Archway Road, London, UK), 2.7 µL of nuclease-free water, and 1 µL of RNA.

After reaction setup, the samples were subjected to amplification in a real-time thermal cycler. The cycling conditions were as follows: 50 °C for 10 min, 95 °C for 2 min, followed by 40 cycles of 95 °C for 5 s and 60 °C for 20 s. All reactions were performed in triplicate for each sample.

Melting curve analysis confirmed that each reaction amplified a single specific PCR product. Each analysis plate included eight genes: three reference genes and five target genes, with three samples run in triplicate for each gene, in addition to a no-template control (NTC).

Relative gene expression values for the target genes were determined using the 2^−ΔΔCt^ method [27]. Results were expressed in arbitrary units and normalized to the reference genes selected: *TBP* (TATA-box binding protein), *RPL4* (ribosomal protein L4), and *B2M* (beta-2-microglobulin). The selection of these genes was based on literature data and the stability of their expression in both analyzed tissues. Additionally, the control group consisted of randomly selected females from the D line, allowing comparison of gene expression levels among the different experimental groups. The geometric mean of the Ct (threshold cycle) values of the reference genes was calculated as described by Vandesompele et al. (2002) [28].

### 2.7. Halothane Genotyping

Genotyping for the detection of mutations in the Halothane gene (RYR1) was performed using genomic DNA extracted from muscle tissue samples. Approximately 50 mg of tissue was placed in microtubes and incubated in a dry bath at 37 °C for 10 min. Subsequently, 290 µL of lysis buffer was added, composed of 246.5 µL of nuclease-free water, 29 µL of 10% SDS, 5.8 µL of 0.5 M EDTA, and 8.7 µL of 1 M Tris-HCl, along with 5 µL of proteinase K. The mixture was homogenized and incubated in a water bath at 55 °C for 16 h (overnight).

Subsequently, 100 µL of 5 M NaCl was added, followed by additional homogenization. The solution was then centrifuged at 13,000 rpm for 10 min, and the supernatant was transferred to a new microtube containing 600 µL of absolute ethanol (100%). After centrifugation for 40 min at 12,000 rpm, the supernatant was discarded, and the pellet was washed with 600 µL of 70% ethanol. A second centrifugation was performed under the same conditions, and after discarding the supernatant, the microtubes were placed in a dry bath at 37 °C for 40 min to allow complete drying of the material. The precipitated DNA was eluted in 50 µL of nuclease-free water and stored at −20 °C until further analyses.

Genotyping was performed according to the method described by Fujii et al. (1991) [29], based on polymerase chain reaction–restriction fragment length polymorphism (PCR-RFLP). The halothane gene presents three possible genotypes: homozygous dominant, heterozygous, and homozygous recessive, with the recessive allele being associated with lower meat quality due to the presence of the mutation.

### 2.8. Statistical Analysis

Statistical analyses were conducted using RStudio software (version 4.3.1) for meat quality data and SAS software v. 9.4 for gene expression data. A completely randomized design was adopted in a 3 × 2 factorial arrangement, considering three genetic lines (D, H, and P) and two sexes (IM and females). Each pig was treated as an individual experimental unit.

For the analysis of phenotypic traits and gene expression variables in muscle and adipose tissues, a general linear model (GLM) with a crossed classification was applied, considering the fixed effects of genetic line (D, H, or P), sex (IM or female), and their interaction (line × sex), as well as the random residual effect. The assumptions of the analysis of variance models (normality and homogeneity of residuals) were simultaneously verified using studentized residual analyses.

Before analysis, the data were screened for outliers, which were subsequently removed. The residuals of each variable were tested for normality using the Shapiro–Wilk test, and only data that met the assumption of normality were included in further analyses. When significant fixed effects were detected in the Type III ANOVA, Tukey’s test was appropriately used for mean comparisons to maintain the joint confidence level at 5% significance (*p* ≤ 0.05).

## 3. Results

### 3.1. Pork Quality

The interaction between genetic line and sex was significant for tenderness (*p* = 0.02) and for the instrumental color parameters L* and b* (*p* = 0.05) (Table 1). According to the analysis of tenderness measured by Warner-Bratzler shear force (WBSF) (Table 2), immunocastrated males from the D line exhibited higher WBSF values, indicating tougher meat, whereas females from the same genetic line showed lower values, corresponding to more tender meat.

Regarding the breakdown of instrumental color parameters (Table 3), immunocastrated males from the H line had higher L* values, indicating lighter-colored meat compared to females of the same line. On the other hand, females from the D and P lines exhibited more yellowish meat than immunocastrated males, as indicated by higher b* values (Table 4).

Regarding the main effects, only the genetic line was significant for drip loss and intramuscular fat content (Table 1). Drip loss was higher (*p* = 0.03) in meat from animals of the H line compared to the P line, while the D line did not differ significantly from the others. Conversely, the P line showed higher intramuscular fat content, given by the ether extract, whereas the H line had the lowest values (*p* = 0.04); no significant differences were observed for the D line compared to the others. Furthermore, no significant effects of genetic line were detected on cooking loss, moisture, a* values, pH at 1h and 24h postmortem, final pH, subjective color score, or marbling score (*p* > 0.05)

### 3.2. Gene Expression

**Figure 2:**
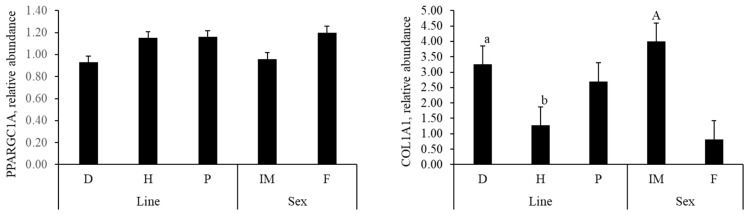

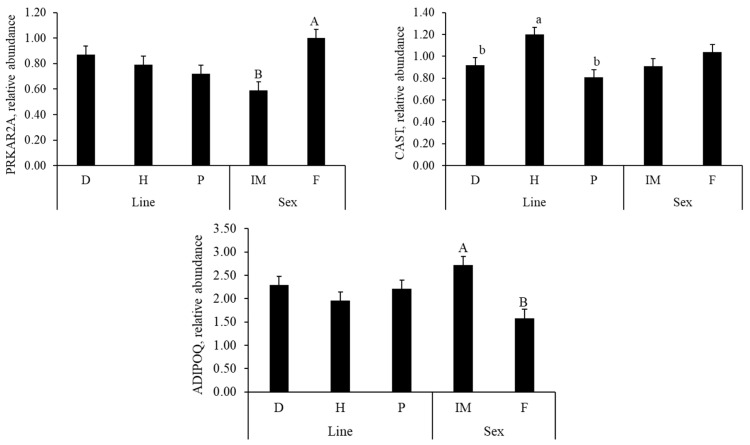
Relative abundance of PPARG coactivator 1 alpha (*PPARGC1A*), collagen type I alpha 1 chain (*COL1A1*), protein kinase cAMP-dependent type II regulatory subunit alpha (*PRKAR2A*), calpastatin (*CAST*), and adiponectin, C1Q and collagen domain containing (*ADIPOQ*) in muscle tissue of pigs from different paternal lines (D, H, and P) and sexes (IM and F). Line D—½ Duroc × ½ DB90; Line H—½ (Duroc and Pietrain) × ½ DB90; Line P—½ Pietrain × ½ DB90. Sex category: IM = Immunocastrated males; F = Female. Values are the means ± SEM. a,b: Within genetic lines, means bearing different letters differ (*p* < 0.05). A,B: Within sex category, means bearing different letters differ (*p* < 0.05).

There was no interaction between line and sex for the expression of any genes in muscle tissue (Figure 2). The expression of the *COL1A1* gene was affected by line (*p* = 0.03), with pigs from both lines D and P showing higher *COL1A1* expression compared to those from line H. Additionally, the genes *COL1A1* (*p* < 0.01) and *PRKAR2A* (*p* = 0.04) were also affected by sex, with IM displaying lower relative expression of *PRKAR2A* compared to females, whereas females exhibited lower *COL1A1* expression than IM. The expression of the *CAST* gene was also influenced by line (*p* = 0.04), with pigs from the line H showing higher abundance of this gene compared to those from lines D and P.

**Figure 3:**
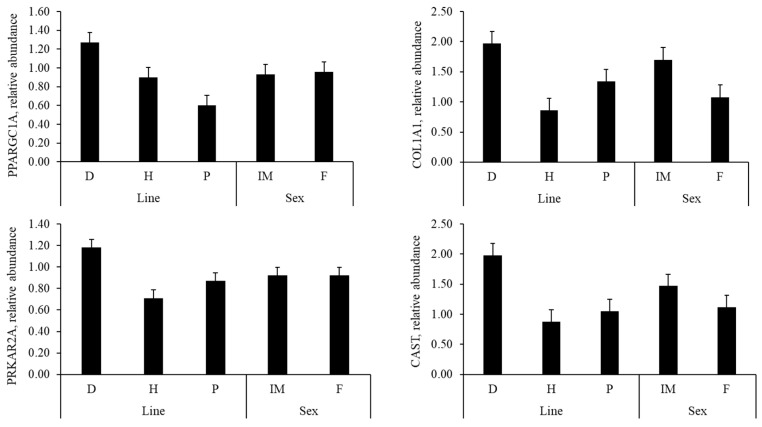

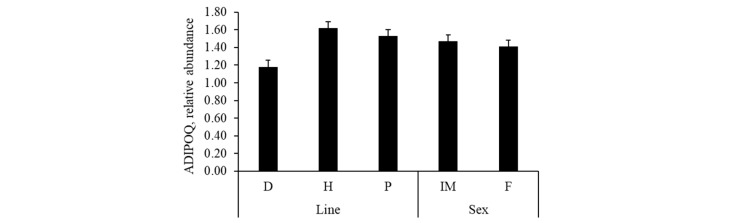
Relative abundance of PPARG coactivator 1 alpha (*PPARGC1A*), collagen type I alpha 1 chain (*COL1A1*), protein kinase cAMP-dependent type II regulatory subunit alpha (*PRKAR2A*), calpastatin (*CAST*), and adiponectin, C1Q and collagen domain containing (*ADIPOQ*) in adipose tissue of pigs from different paternal lines (D, H, and P) and sexes (IM and F). Line D—½ Duroc × ½ DB90; Line H—½ (Duroc and Pietrain) × ½ DB90; Line P—½ Pietrain × ½ DB90. Sex category: IM = Immunocastrated males; F = Female. Values are the means ± SEM.

There was no interaction between line and sex for the relative gene expression in adipose tissue (Figure 3). There was no main effect of sex on gene expression. However, the expression of the *CAST* gene was influenced by line (*p* = 0.04), with pigs from line D showing higher relative abundance (*p* < 0.05) than lines H and P.

### 3.3. Halothane Genotyping

Based on genotyping performed to detect the mutation in the Halothane gene, the HAL^Nn^ genotypes were identified in the population of the present study. All animals analyzed presented the heterozygous HAL^Nn^ genotype, which is associated with increased resistance to the adverse effects related to the mutation in this gene.

## 4. Discussion

It is well established that immunocastration offers a practical, welfare-friendly, and sustainable alternative to surgical castration, providing benefits in terms of feed efficiency and pork quality as well [30,31]. However, it is important to note that immunocastration alters the hormonal profile and causes associated physiological effects, including growth, body composition, and behavior [32], which may attenuate the naturally sex-related biological differences. Raising entire males in Brazil is legal but faces management and market challenges, resulting in a growing focus on the adoption of immunocastration in commercial pig production. Therefore, our study was designed to evaluate whether different genetic lines influence pork quality and candidate gene expression in both immunocastrated pigs and females.

### 4.1. Pork Quality

The selection of breed for genetic crossbreeding plays a fundamental role in the production process, directly influencing the quality of the final product. This choice is driven by market demands, which are aligned with consumer expectations and meat quality criteria [33]. In this context, the analysis of pork meat among the different paternal lines D, H, and P revealed significant differences that impact product quality. Furthermore, significant differences were also observed with respect to sex (IM and females), as well as in the interaction between genetic line and sex. Understanding how these phenotypic differences intersect with genetic factors remains a relatively unexplored area, particularly in commercial pig lines. The present work advances this field by linking production traits with gene expression patterns, thus bringing additional knowledge that can strengthen scientific research and breeding strategies.

It is well established that certain genes stand out for their marked influence on meat quality. Among the genetic factors that influence pork meat quality, particular attention has been given to the ryanodine receptor 1 (RYR1), due to its association with porcine stress syndrome and PSE (pale, soft, and exudative) meat characteristics [34]. In this study, all animals were heterozygous (HALNn) for the Halothane gene, a genotype known to confer greater resistance to stress-related negative effects, without compromising meat quality traits. This genetic background may have contributed to the results observed in meat quality across the different paternal lines and sexes by minimizing the occurrence of stress-induced quality defects [34].

Pork color stands out as one of the main factors influencing consumer acceptance. In our study, the L* parameter, associated with meat lightness, was significantly higher in IM compared to females from line H. The lighter appearance of the meat may be related to the content of water present in the muscle tissue, as meat with lower water-holding capacity reflects light more diffusely, resulting in a paler appearance [35]. However, our findings demonstrated that both IM and females exhibited similar drip loss values, which contradicts the expected relationship that lower water retention would result in increased meat lightness [35].

Although IM exhibited darker meat compared to females, the differences in loin color were not visually noticeable, given that the subjective color evaluation did not reveal significant differences, which is consistent with the study of Gispert et al. (2010) [8]. This highlights the importance of instrumental color measurements, as visual perception may be subjective.

In contrast to previous studies [7,8,35], the present study demonstrated that sex alone had a significant effect on the b* parameter, with females exhibiting meat with a more pronounced yellow hue compared to IM. This result indicates a potential disadvantage for females in terms of meat color quality, as a more yellowish appearance is often perceived less favorably by consumers and may negatively impact purchasing decisions.

Meat yellowing is directly linked to intramuscular fat content, as higher levels of marbling tend to result in increased b* values due to the presence of liposoluble pigments such as carotenoids, which are responsible for the yellowish coloration [36]. However, in our study, intramuscular fat content was similar between sexes, which is further supported by the lack of significant differences observed in the subjective marbling evaluation.

Another highly relevant parameter in pork quality is drip loss, which refers to the meat’s ability to retain water and directly affects meat weight, appearance, and tenderness. Drip loss is closely related to muscle structure and the integrity of myofibrillar proteins, which are influenced by genetic factors, management conditions, and post-mortem metabolism [37,38].

The high drip loss observed in line H represents a significant challenge for the pork industry, as it reduces the nutritional value through losses of soluble proteins, minerals, and vitamins, and can also result in a product with a drier texture that is less appealing to consumers [26,37]. Furthermore, the excess water accumulated in packaging because of the exudation may negatively impact consumer perception of the product.

We observed that line P exhibited a higher intramuscular fat content, whereas line H showed lower values for this parameter. As intramuscular fat acts as a physical barrier against exudation, these results may partly be accounted for by the lower drip loss in line P observed herein. Thus, regarding drip loss, the meat from pigs of line P demonstrated more favorable characteristics for both premium products and fresh meat commercialization.

In addition to its influence on water retention, intramuscular fat is related to the sensory quality of pork, directly affecting traits such as tenderness and flavor [39,40]. The higher intramuscular fat deposition observed in line P may be beneficial for product acceptance by consumers, as it is commonly associated with juicier and more flavorful meat [41]. Conversely, the lower intramuscular fat content in line H may result in meat with a firmer texture and reduced juiciness, thereby being less desirable in certain markets [41]. However, although intramuscular fat content is traditionally associated with meat tenderness, our study did not demonstrate a significant relationship between these variables across the evaluated lines.

Tenderness, in addition to being influenced by intramuscular fat content, is also affected by the degradation of myofibrillar proteins during post-mortem aging [42]. In the present study, IM from line D exhibited higher shear force values, indicating tougher meat, whereas females of the same line showed more tender meat. Thus, females from this line demonstrated advantages regarding this quality parameter, as consumers tend to prefer more tender meat [43].

### 4.2. Gene Expression Profile

The analysis of gene expression of key genes associated with pork quality across different lines, such as D, H, and P, and sexes (females and IM), allows for a more detailed understanding of the factors influencing the physicochemical attributes of the meat. The five candidate genes selected for this study (*ADIPOQ*, *CAST*, *COL1A1*, *PPARGC1A*, and *PRKAR2A*), known to be involved in metabolic processes, including muscle development and fat deposition, were analyzed to evaluate how their expression varies among lines and sexes.

The appropriate selection of reference genes is essential to ensure accuracy and reliability in gene expression analyses. In our study, the genes *B2M*, *TBP*, and *RPL4* were selected as internal controls, i.e., housekeeping genes, because of their stability in muscle and subcutaneous adipose tissues as reported in previous studies [11,44,45,46]. The use of these reference genes enabled reliable analysis of the expression of target genes such as *COL1A1*, *PPARGC1A*, *CAST*, *ADIPOQ*, and *PRKAR2A*, which still have limited studies related to pork quality [13,47,48].

Calpastatin (*CAST*), as an endogenous inhibitor of calpains, plays a fundamental role in regulating post-mortem muscle proteolysis, directly influencing meat tenderness [24,25]. The *CAST* activity is associated with the degradation of muscle proteins, an important process for the tenderization of muscle tissue after slaughter [24].

The higher expression of the *CAST* gene observed in line H in our study suggests a more intense inhibition of calpains in the muscle tissue of these animals. Elevated expression of this gene implies greater inhibitory activity on calpains, thereby reducing protein degradation and resulting in lower tenderness [25]. However, this relationship was not observed in the current study, as the genetic lines exhibited similar tenderness values.

Furthermore, significant expression of the *CAST* gene has been previously associated with drip loss [11]. The inhibition of calpains immediately post-mortem reduces the degradation of structural proteins such as integrins, which limits the formation of drip channels and thus increases water loss in fresh meat [11,26].

Consequently, higher CAST expression in pork muscle is associated with increased drip loss because the greater stability of cellular structures prevents the formation of spaces that normally help retain water within the muscle tissue [11,26]. Our results indicate that line H exhibited higher *CAST* gene expression and, consequently, greater drip loss in the meat. In contrast, line P and D showed lower *CAST* expression and, correspondingly, lower drip loss.

In adipose tissue, *CAST* gene expression was also influenced by the genetic line. However, unlike the observations in muscle, line H exhibited lower expression of this gene, whereas line D showed the highest expression. This result suggests that *CAST* expression in adipose tissue is not related to drip loss, since the calpain–calpastatin system plays a predominant role in postmortem proteolysis within muscle fibers rather than in adipose tissue [11,26,49].

In muscle, calpastatin regulates calpain activity, affecting the degradation of cytoskeletal proteins such as titin and desmin, which directly influence tenderness and water-holding capacity [26]. In adipose tissue, however, *CAST* expression is more likely associated with cell turnover and lipid metabolism rather than structural protein breakdown [49].

The calpain-calpastatin system plays a critical role in postmortem proteolysis within muscle fibers, directly influencing tenderness and water-holding capacity. However, in adipose tissue, this system is primarily involved in cell turnover and lipid metabolism, with minimal relevance to the postmortem process [11].

Similarly, the *COL1A1* gene, which encodes the alpha-1 chain of type I collagen, plays an important role in collagen structure formation and cell adhesion [50]. The expression of this gene is associated with the formation of the extracellular matrix and, consequently, with the water retention capacity of muscle tissue [51].

McBryan et al. (2010) [11] reported that the *COL1A1* gene is associated with drip loss in pork. The authors observed that increased expression of this gene may be linked to higher drip loss, as it enhances type I collagen synthesis, resulting in a denser and more rigid extracellular matrix, thereby reducing the muscle tissue’s ability to retain water.

In our study, a significant difference in the expression of this gene was observed between the sexes, with IM exhibiting higher *COL1A1* expression. However, this difference in gene expression did not translate into variations in drip loss, contradicting the expected relationship between these parameters. This suggests that, despite *COL1A1’s* influence on collagen structure, its expression was insufficient to significantly impact meat exudation in the animals evaluated.

Furthermore, *COL1A1* gene expression in muscle was also influenced by genetic lineage. Although this gene has been associated with drip loss [11], our results indicated the opposite, as lineage H, which showed lower *COL1A1* expression, exhibited higher drip loss compared to the other lineages.

In contrast to the expression of the *COL1A1* gene in muscle tissue, the *PRKAR2A* gene exhibited higher expression levels in females compared to IM. The *PRKAR2A* is an important molecule in cellular signaling, playing a key role in regulating metabolism and cell growth in mammals [13].

Elevated expression of *PRKAR2A* in pigs has several relevant implications for meat quality, especially concerning intramuscular fat deposition. Previous studies have reported a direct relationship between higher expression of this gene and increased intramuscular fat, which is associated with improved marbling and sensory characteristics of the meat [44,52]. However, our results did not show significant differences in intramuscular fat content between the sexes evaluated.

Interestingly, although the *PRKAR2A* gene is known to influence fat deposition in pigs, no significant effects of genetic lines, sex, or their interaction were observed on the expression of this gene in adipose tissue samples from the animals studied.

The higher expression of PRKAR2A observed in females is consistent with its established role in regulating lipid metabolism. This gene encodes a regulatory subunit of protein kinase A (PKA), a key enzyme involved in lipolysis and lipid storage processes. Sex-related hormonal differences observed herein corroborate a previous study in mice, which demonstrated sex differences in PKA signaling regulation [53]. Despite this transcriptional difference, no phenotypic variation in intramuscular fat content was detected, suggesting that post-transcriptional or post-translational regulatory mechanisms may buffer these effects at the phenotypic level. Alternatively, the expression difference might reflect subtle metabolic distinctions [54] not captured by the static measurement of fat content but relevant to lipid turnover dynamics in muscle tissue.

These findings are relevant not only for the quality of the final product but also for sustainability in meat production. Understanding the interactions between gene expression, quality traits, and production factors can contribute to the selection of more efficient lines that promote reduced waste and losses during processing. Furthermore, genetic improvement focused on traits such as reduced drip loss and improved color, for example, may enhance consumer acceptance of meat or meat products, thereby reducing environmental impact by maximizing yield. Thus, integrating aspects of quality and sustainability can benefit the production chain in meeting the demands for more responsible and sustainable meat production.

## 5. Conclusions

This study evaluated the influence of sex and genetic line on pork meat quality traits and the expression of related genes, including *COL1A1*, *CAST*, *PPARGC1A*, *PRKAR2A*, and *ADIPOQ*, in both muscle and adipose tissues. The analysis of the expression of these quality-related genes contributed to a better understanding of the mechanisms affecting attributes such as color, drip loss, and tenderness, while also highlighting the critical role of genetic line and sex in the regulation of these processes.

Regarding genetic lines, meat from line H exhibited greater drip loss and lower intramuscular fat content, associated with higher *CAST* expression, suggesting a potential link between proteolysis regulation and water-holding capacity. In contrast, line P showed higher intramuscular fat deposition, a trait commonly associated with enhanced juiciness, flavor, and consumer acceptability. These differences highlight the influence of genetic background on both water-holding capacity and fat deposition, two key determinants of pork meat quality. In adipose tissue, genetic line also affected *CAST* expression, although without corresponding changes in meat quality traits, indicating tissue-specific regulatory mechanisms.

Concerning sex effects, significant sex-related differences and line × sex interactions were also observed. Immunocastrated males from line D produced tougher meat, whereas females from the same line exhibited greater tenderness, reinforcing the role of sex effect in modulating meat texture. Moreover, instrumental color parameters revealed subtle variations not detectable by visual assessment, emphasizing the value of objective colorimetry as a reliable tool for evaluating pork quality. At the molecular level, PRKAR2A expression was higher in females, consistent with its role in lipid metabolism, although no phenotypic differences in intramuscular fat content were detected.

These findings improve our understanding of the molecular mechanisms underlying meat quality and highlight tissue-specific regulatory effects. Future studies should further investigate the pathways linking gene expression to phenotypic traits and explore how these insights can be applied in breeding programs to enhance pork quality and consumer satisfaction.

## Figures and Tables

**Table 1:** Effects of different terminal sire lines (D, H, and P) and sex on meat quality parameters in the *Longissimus lumborum* muscle of pigs.

Item	Line ^1^			Sex ^2^		Pooled SEM ^3^	*p*-Value		
	D	H	P	IM	F		Line	Sex	Line × Sex
Moisture (%)	73.82	74.13	73.34	73.84	73.70	1.31	0.15	0.70	0.96
Drip loss (%)	3.20 ^a,b^	3.60 ^a^	2.61 ^b^	3.06	3.21	1.19	0.03	0.59	0.17
Intramuscular fat (%)	2.17 ^a,b^	1.78 ^b^	2.55 ^a^	2.03	2.28	0.97	0.04	0.32	0.36
a* ^4^	4.74	5.19	5.10	4.97	5.04	0.95	0.26	0.76	0.80
pH at 1 h	6.44	6.44	6.47	6.46	6.44	0.17	0.84	0.58	0.63
pH at 24 h	6.03	6.05	6.08	6.07	6.04	0.24	0.80	0.57	0.78
Final pH ^5^	5.66	5.64	5.68	5.64	5.68	0.11	0.45	0.19	0.77
Visual color score	3.07	3.28	3.25	3.12	3.27	0.45	0.22	0.20	0.16
Marbling score	1.75	1.77	1.88	1.84	1.76	0.54	0.69	0.56	0.80
Cooking loss (%)	26.63	26.93	25.91	26.37	26.62	3.21	0.57	0.74	0.31

^1^ Different paternal lines: Line D—½ Duroc × ½ DB90; Line H—½ (Duroc and Pietrain) × ½ DB90; Line P—½ Pietrain × ½ DB90. ^2^ Sex category: IM = Immunocastrated males; F = Female. ^3^ Pooled SEM: Combined standard error of the mean. ^4^ Instrumental color parameters: red/green coordinate (a*); ^5^ Final pH after storage and thawing. ^a,b^ Means followed by different letters in the same row, by genetic line and sex, differ significantly (*p* ≤ 0.05).

**Table 2:** Breakdown of the interaction between line and sex for Warner-Bratzler shear force (WBSF) (*p* = 0.02).

Line ^1^	Sex ^2^			
	IM		F	
	Mean	SE ^3^	Mean	SE
Line D	±4.83 ^a^	0.26	±3.92 ^b^	0.25
Line H	±4.61 ^a^	0.26	±5.16 ^a^	0.26
Line P	±4.56 ^a^	0.27	±5.16 ^a^	0.26

^1^ Different paternal lines: Line D—½ Duroc × ½ DB90; Line H—½ (Duroc and Pietrain) × ½ DB90; Line P—½ Pietrain × ½ DB90. ^2^ Sex category: ^3^ Standard error. IM = Immunocastrated males; F = Female. ^a,b^ Means followed by different letters in the same row, by genetic line and sex, differ significantly (*p* ≤ 0.05).

**Table 3:** Breakdown of the interaction between line and sex for the color parameter L* (*p* = 0.05).

Line ^1^	Sex ^2^			
	IM		F	
	Mean	SE ^3^	Mean	SE
Line D	±51.00 ^a^	0.62	±51.4 ^a^	0.57
Line H	±52.40 ^a^	0.62	±50.20 ^b^	0.60
Line P	±51.10 ^a^	0.63	±51.30 ^a^	0.60

^1^ Different paternal lines: Line D—½ Duroc × ½ DB90; Line H—½ (Duroc and Pietrain) × ½ DB90; Line P—½ Pietrain × ½ DB90. ^2^ Sex category: IM = Immunocastrated males; F = Female. ^3^ Standard error. ^a,b^ Means followed by different letters in the same row, by genetic line and sex, differ significantly (*p* ≤ 0.05).

**Table 4:** Breakdown of the interaction between line and sex for the color parameter b* (*p* = 0.05).

Line ^1^	Sex ^2^			
	IM		F	
	Mean	SE ^3^	Mean	SE
Line D	±5.33 ^b^	0.28	±6.17 ^a^	0.27
Line H	±5.76 ^a^	0.28	±5.44 ^a^	0.28
Line P	±5.40 ^b^	0.29	±6.31 ^a^	0.28

^1^ Different paternal lines: Line D—½ Duroc × ½ DB90; Line H—½ (Duroc and Pietrain) × ½ DB90; Line P—½ Pietrain × ½ DB90. ^2^ Sex category: IM = Immunocastrated males; F = Female. ^3^ Standard error. ^a,b^ Means followed by different letters in the same row, by genetic line and sex, differ significantly (*p* ≤ 0.05).

## Data Availability

The original contributions presented in this study are included in the article/Supplementary Materials. Further inquiries can be directed to the corresponding author.

## Supplementary Materials

The following supporting information can be downloaded at: https://www.mdpi.com/article/10.3390/ani15233363/s1, Table S1: Gene, gene names, primer sequences (F: forward primer; R: reverse primer), amplified fragment size expressed in base pairs (bp), GenBank accession number (Gene ID) from NCBI, and references.
