# Supplementary material for: Pork Quality and Expression of Genes Involved in Muscularity and Fat Deposition in Different Commercial Lines and Sexes of Pigs
Source: Animals (Basel). 2025 Nov 21;15(23):3363. doi: 10.3390/ani15233363 (PMC12691475; doi:10.3390/ani15233363)
Supplement: Supplementary file 1 [file animals-15-03363-s001.zip › animals-3943032-supplementary.pdf]

Table S1. Gene, gene names, primer sequences (F: forward primer; R: reverse primer), amplified fragment size expressed in base pairs (bp), GenBank accession number (Gene ID) from NCBI, and references.

**Gene**	**Gene name**	**Primer sequence (5' → 3')**	**Fragment size (bp)**	**Gene ID**	**References**
TBP	TATA-box binding protein	F: TTAATGGTGGTGTTGTGGACGGC<br>R: CCAAATAGCAGCACAGTACGAGCAA	168	110259740	Hamill et al. (2012); McBryan et al. (2010)
RPL4	Ribosomal protein L4	F: AGAGATCCAAAGAGCCCTCCGC<br>R: GCCTGGCGAAGAATGGTGTTTC	144	100038029	Hamill et al. (2012); McBryan et al. (2010)
B2M	Beta-2-microglobulin	F: AAACGGAAAGCCAAATTACC<br>R: ATCCACAGCGTTAGGAGTGA	178	110255236	Hamill et al. (2012); McBryan et al. (2010)
PPARGC1A	PPARG coactivator 1 alpha	F: GGGATGATGGAGACAGCTATGGTT<br>R: TGCTCTTGGTGGAAGCAGGATCAA	204	397013	Gandolfi et al. (2011); Park et al. (2012)
COL1A1	Collagen type I alpha 1 chain	F: CCAGTCACCTGCGTACAGAA<br>R: ACGTCATCGCACAACACATT	110	100738123	McBryan et al. (2010)
PRKAR2A	Protein kinase cAMP-dependent type II regulatory subunit alpha	F: CCAATTCCTAGCAGATTTGATCGGCG<br>R: GCATCGAGGACTTGAGAAAGTTGTTCC	197	397493	Park et al. (2012)
CAST	Calpastatin	F: GTGCCTCCTCCAGACACTTC<br>R: CTCGGTTTTCTTCCCATCAG	108	397135	McBryan et al. (2010)
ADIPOQ	Adiponectin, C1Q and collagen domain containing	F: GGAGATACAGGTCTTACTGGTCCTA<br>F: CAGGAATGTTGCAGTGGAATTTGCCA	262	397660	Zequan et al. (2022)
